# Alternative RNA splicing of the MEAF6 gene facilitates neuroendocrine prostate cancer progression

**DOI:** 10.18632/oncotarget.15854

**Published:** 2017-03-02

**Authors:** Ahn R. Lee, Yinan Li, Ning Xie, Martin E. Gleave, Michael E. Cox, Colin C. Collins, Xuesen Dong

**Affiliations:** ^1^ Vancouver Prostate Centre, Department of Urologic Sciences, The University of British Columbia, Vancouver V6H 3Z6, Canada

**Keywords:** MEAF6, RNA splicing, tumor progression, neuroendocrine prostate cancer

## Abstract

Although potent androgen receptor pathway inhibitors (ARPI) improve overall survival of metastatic prostate cancer patients, treatment-induced neuroendocrine prostate cancer (t-NEPC) as a consequence of the selection pressures of ARPI is becoming a more common clinical issue. Improved understanding of the molecular biology of t-NEPC is essential for the development of new effective management approaches for t-NEPC. In this study, we identify a splice variant of the MYST/Esa1-associated factor 6 (MEAF6) gene, MEAF6-1, that is highly expressed in both t-NEPC tumor biopsies and neuroendocrine cell lines of prostate and lung cancers. We show that MEAF6-1 splicing is stimulated by neuronal RNA splicing factor SRRM4. Rather than inducing neuroendocrine trans-differentiation of cells in prostate adenocarcinoma, MEAF6-1 upregulation stimulates cell proliferation, anchorage-independent cell growth, invasion and xenograft tumor growth. Gene microarray identifies that these MEAF6-1 actions are in part mediated by the ID1 and ID3 genes. These findings suggest that the MEAF6-1 variant does not induce neuroendocrine differentiation of prostate cancer cells, but rather facilitates t-NEPC progression by increasing the proliferation rate of cells that have acquired neuroendocrine phenotypes.

## INTRODUCTION

Next generation androgen receptor (AR) pathway inhibitors (ARPIs) prolong survival of patients with metastatic castrate resistant prostate cancer (CRPC) [1, 2]. However, a consequence of the selection pressures exerted by the more potent ARPIs is that they can promote the emergence of AR-independent CRPC, one variant of which is treatment-induced neuroendocrine prostate cancer (t-NEPC) [3–6]. t-NEPC is reported in 25–30% of post-ARPI CRPC patients and the rate of occurrence is predicted to rise with the widespread use of ARPIs [7]. Once diagnosed, the average survival of t-NEPC patients is only ~7 months [8]. No targeted therapy is available for t-NEPC patients, only systemic chemotherapy regimens, reflecting our limited knowledge on the molecular underpinnings of t-NEPC progression.

There is accumulating evidence indicating that t-NEPC is clonally derived from adenocarcinoma (AdPC) precursors [3, 9]. Whole exome sequencing has shown similar mutational landscapes between t-NEPC and AdPC [3, 5, 10]. Case studies reveal that cell populations of AdPC, AdPC with neuroendocrine differentiation, and t-NEPC co-exist in the same tumor [11]. Intermediate morphological and phenotypical transitions exist in cancer cells between the boundaries of AdPC and t-NEPC cell populations, indicating dynamic neuroendocrine differentiation processes. The transition of AdPC to t-NEPC has been reported in patient derived xenografts (PDXs) following castration, with no genotypic alterations pre- and post-castration [5]. Moreover, in castrated mice, when neural RNA splicing factor SRRM4 is introduced exogenously, LNCaP AdPC cells can be transformed into t-NEPC xenografts [4]. Additionally, introduction of exogenous N-MYC into primary basal epithelial cells can induce NEPC xenografts [12]. These findings collectively support that t-NEPC originates from AdPC.

The transition of AdPC to t-NEPC is a complicated process that may involve both cell differentiation and proliferation, two distinguishable and coordinated processes controlled by multiple genes. Luminal epithelial AR signaling is critical in the maintenance of epithelial cell differentiation in the adult prostate [13]. As a result, AR inhibition can trigger prostate cancer cell de-differentiation to confer adaptive plasticity and promote phenotype reprogramming. While AR blockade is necessary for t-NEPC establishment, it is insufficient since only about 25, 30% ARPI-treated tumors are transformed into t-NEPC [7]. Similarly, AdPC cells can acquire a neuroendocrine phenotype when treated with IL-6 or cAMP [14, 15], but these cells have not been reported to be capable of forming a t-NEPC tumor. While AdPC with neuroendocrine differentiation can be found in 30, 100% of AdPC [16, 17], expression of neuroendocrine markers is not sufficient for t-NEPC establishment. To establish a t-NEPC tumor, the cells that express neuroendocrine markers must gain a proliferative state for clonal expansion. Targeting genes that facilitate NEPC cell proliferation would be more effective in delaying t-NEPC progression, particularly in patients under ARPI treatment.

Using whole-transcriptome sequencing of AdPC and NEPC patient biopsies from the Beltran [18] and Vancouver Prostate Centre (VPC) [19] cohorts, we reported a NEPC-unique RNA splicing signature that is predominantly driven by SRRM4. SRRM4 can transform LNCaP AdPC cells into NEPC xenografts, suggesting that SRRM4 regulates RNA splicing of multiple genes that are responsible for neuroendocrine differentiation and cell proliferation processes during t-NEPC progression. In fact, we have shown that SRRM4 regulates RNA splicing of the REST gene and re-programs its functions to confer a neuroendocrine phenotype in AdPC cells [4]. However, REST knockdown in LNCaP cells does not allow a t-NEPC xenograft to develop, supporting the notion that SRRM4-mediated REST gene splicing leads to neuroendocrine differentiation but does not induce proliferation sufficient for clonal expansion and t-NEPC tumor establishment. In searching for other SRRM4-targeted RNA splicing that may contribute to NEPC progression, we have found a highly expressed splice variant of the MYST/Esa1-associated factor 6 (MEAF6) gene, MEAF6-1, in NEPC tumor samples. Although a protein component of the histone acetyltransferase (HAT) complexes [20, 25], there have been no studies on the cellular functions of MEAF6. MEAF6-1 and MEAF6-2 are two protein coding transcripts from the MEAF6 gene, whereby exon 6 is included in MEAF6-1 but skipped in MEAF6-2 mRNA. Since increased MEAF6-1 expression is closely associated with NEPC, we set out to determine whether MEAF6-1 contributes to NEPC progression.

## RESULTS

### RNA splicing of the MEAF6 gene is associated with NEPC progression

**Figure 1:**
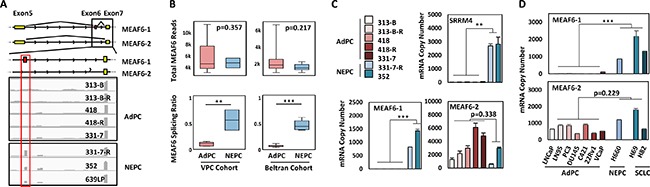
RNA splicing of the MEAF6 gene is associated with NEPC progression (**A**) Illustration of MEAF6-1 and MEAF6-2 RNA. The alternatively spliced exon (exon 6) is illustrated in red, where constitutive exons are denoted in yellow. Integrative Genomics Viewer (IGV) was used to visualize the coverage of MEAF6 by RNA-seq reads in AdPC and NEPC patient tumors and patient-derived xenografts (PDXs). Grey areas represent the sequencing depth of the respective exon, where the more prominent peaks reflect the significant presence of the positioned exon. (**B**) MEAF6 splicing ratio (MEAF6-1:MEAF6-2 RNA-seq reads per base-pair) and MEAF6 total expression obtained from RNA-seq data of AdPC and NEPC patient tumor samples (NEPC *n* = 5 and AdPC *n* = 8 in VPC cohort; NEPC *n* = 6 and AdPC *n* = 32 in Beltran cohort) (**C**) Validation of RNA-seq data, Figure 1A, using real-time qPCR on RNA isolated from AdPC and NEPC PDX. (**D**) Profiling of mRNA copy numbers of MEAF6 splice variants in a panel of AdPC cell lines (LNCaP, LN95, PC3, DU145, C421, 22Rv1 and VCaP) and NEPC cell line (NCI-H660) as well as small cell lung cancer (SCLC; NCI-H69 and -H82), which is a neuroendocrine cancer of the lung. This was done via real-time qPCR for absolute quantification of total MEAF6-1 and MEAF6-2 using a standard curve. All results are presented as the mean ± SEM (Student *t-test* ***denotes *p <* 0.001 and **denotes *p <* 0.01). AdPC, adenocarcinoma prostate cancer; NEPC, neuroendocrine prostate cancer; VPC, Vancouver Prostate Centre; SCLC, small cell lung cancer.

Whole transcriptome sequencing data on AdPC and NEPC tumor samples from the VPC (8 AdPC and 5 NEPC samples) and Beltran (32 AdPC and 6 NEPC samples) cohorts were analyzed by a computational tool, COMPAS [4], which identified an increase in the ratio of MEAF6-1:MEAF6-2 in NEPC patients (*p* = 0.0034 in the VPC cohort and *p* = 0.0002 in the Beltran cohort), while total MEAF6 mRNAs remained unchanged (Figure 1A–1B). These results indicated that MEAF6 RNA splicing is a unique feature of NEPC. Real-time qPCR assays on tumor samples from PDXs further confirmed that MEAF6-1 mRNA levels in NEPC were about 150-fold higher than AdPC (*p* = 0.001), while MEAF6-2 mRNA levels in NEPC were not statistically different between NEPC and AdPC (*p* = 0.338) (Figure 1C). Increased MEAF6 RNA splicing was also positively correlated with elevated SRRM4 mRNA expression in both xenograft (Figure 1C) and clinical CRPC samples (Supplementary Figure 1A). Additionally, MEAF6 RNA splicing activity was positively correlated with REST RNA splicing (Supplementary Figure 1B). These results collectively suggest that SRRM4 may be also be a regulator of MEAF6 gene splicing. In prostate cancer cell lines, MEAF6-1 was more highly expressed in NEPC cell line NCI-H660 as well as small cell lung cancer (SCLC) cell lines NCI-H69 and -H82, which are two lung cancer cell lines with neuroendocrine differentiation, when compared to MEAF6-1 expression levels in AdPC cell lines (*p* = 0.00028). In contrast, MEAF6-2 mRNA levels were not statistically different in AdPC lines from NCI-H660, -H69, and -H82 cell lines (Figure 1D). Further validation of MEAF6 protein expression could not be done because currently available antibodies cannot differentiate MEAF6 splicing variants from each other, and immunoblotting and immunohistochemistry assays were unable to recognize endogenous MEAF6 proteins. Together, these results indicate that up-regulation of the expression of MEAF6-1 splice variant is closely associated with NEPC progression.

### SRRM4 regulates RNA splicing of the MEAF6 gene

**Figure 2:**
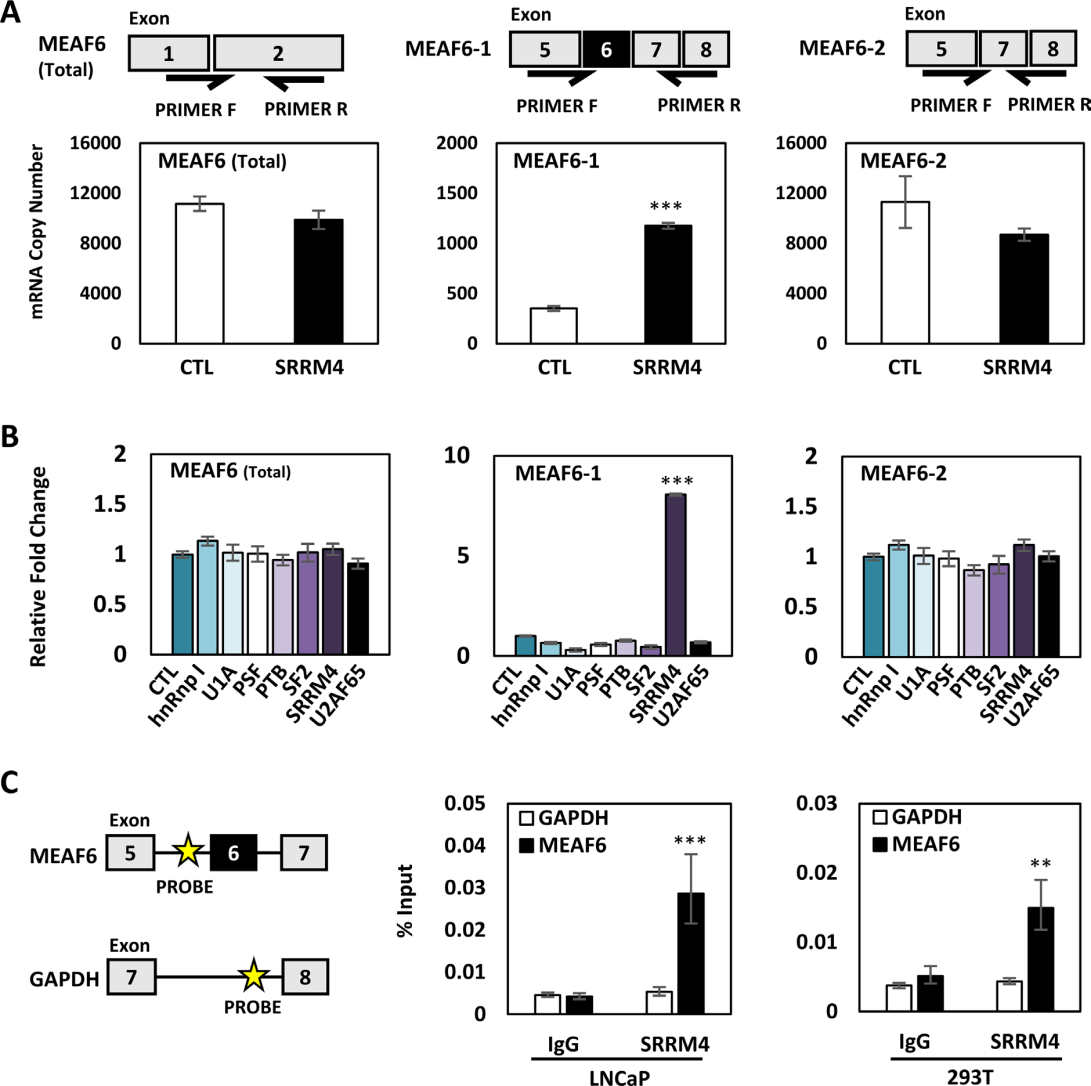
SRRM4 regulates RNA splicing of the MEAF6 gene (**A**) Illustration of splice variant-specific primers designed to detected total MEAF6, MEAF6-1, and MEAF6-2 mRNA levels. LNCaP cells seeded in 6-well plates were transiently transfected with 4 ug of Flag-SRRM4 for 24 hours and were isolated for RNA. Real-time qPCR for absolute quantification of total MEAF6, MEAF6-1, and MEAF6-2 using a standard curve was performed. (**B**) LNCaP cells were transiently transfected with a panel of RNA splicing factors (hnRnp I, U1A, PSF, PTB, SF2, SRRM4, U2AF65). Cells were then extracted for RNA. Relative quantifications of total MEAF6, MEAF6-1, and MEAF6-2 were compared to 18S via real-time qPCR. Results are presented as the mean ± SEM (one-way ANOVA; *n* = 3; ***denotes *p <* 0.001). (**C**) RNA-ChIP was performed with 293T and LNCaP cells in 10 cm dishes. Cells were transfected with 10 ug of Flag-SRRM4 and immunoprecipitated with anti-Flag antibody following fixation with paraformaldehyde. Eluted RNA fragments were used as templates for real-time qPCR primers antisense for intron sequence upstream of alternatively spliced exon of MEAF6-1. Position of probes for MEAF6-1 and GAPDH is indicated by the yellow star. Otherwise indicated, results are presented as the mean ± SEM (Student *t-test*, *n* = 3 ***denotes *p <* 0.001 and **denotes *p <* 0.01).

To determine whether SRRM4 regulates MEAF6 splicing, we transiently transfected SRRM4 expression vector in LNCaP cells. SRRM4 did not alter the levels of total MEAF6 transcripts (Figure 2A). Instead, it induced MEAF6-1 but had no impact on MEAF6-2 mRNA levels. SRRM4 regulation of MEAF6 RNA splicing was further confirmed in SRRM4 knockdown conditions via siRNA (Supplementary Figure 2). To test whether other RNA splicing factors may also regulate MEAF6 gene splicing, we repeated the experiments with a panel of splicing factors and showed that MEAF6 RNA splicing is uniquely regulated by SRRM4 (Figure 2B). Furthermore, RNA chromatin immunoprecipitation (RNA-ChIP) assays confirmed that SRRM4 is recruited to an intron 5 region next to the 3′ splice site for MEAF6-1, but not a control intron region of the GAPDH gene (Figure 2C). In addition, we found that RNA splicing or regulation of MEAF6 mRNA expression was not altered by AR signaling (Supplementary Figure 3). Collectively, these results demonstrate that SRRM4 is an important regulator of MEAF6-1 splicing.

### MEAF6-1 promotes prostate cancer cell growth and invasion

To study the biological functions of MEAF6-1 in prostate cancer cells, we constructed LNCaP and PC-3 cell lines that stably express exogenous MEAF6-1 or MEAF6-2 via lentiviral transduction (Supplementary Figure 4). Neither MEAF6-1 nor MEAF6-2 changed the expression levels of NEPC biomarkers such as CHGA, ENO2, SYP, CHGB and REST, or AdPC biomarkers such as E-Cad (Supplementary Figure 5). These results indicate that neither of the MEAF6 splice variants are involved in neuroendocrine trans-differentiation of prostate cancer cells.

**Figure 3:**
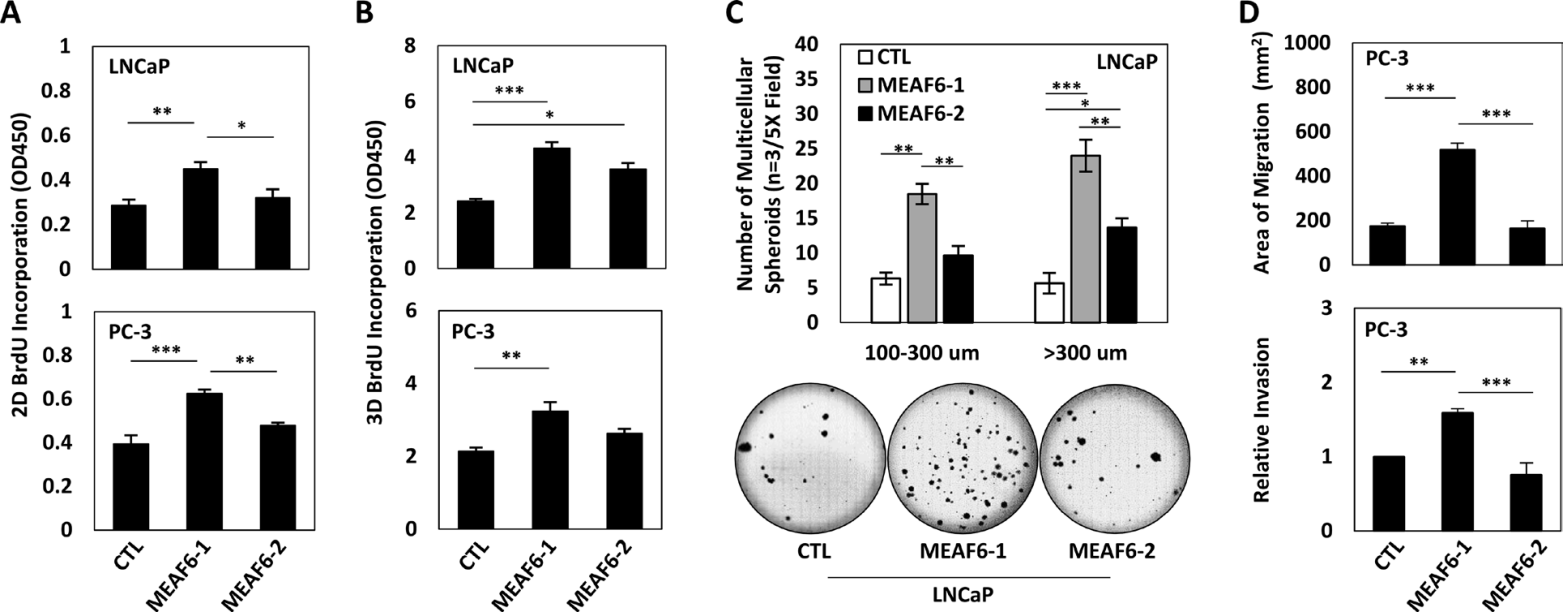
MEAF6-1 promotes prostate cancer cell growth and invasion Using LNCaP or PC-3 cell lines stably expressing control (CTL), MEAF6-1, or MEAF6-2, (**A**) 2D BrdU cell proliferation assays were performed to measure proliferation of cells. BrdU results represent colorimetric quantitative measurements (optical density, OD, at 450 nm wavelength) of cellular BrdU incorporation into DNA. (**B**) Cells were also seeded in matrigel for BrdU proliferation assays to measure the proliferative ability under 3D conditions. (**C**) Soft agar assays were performed on LNCaP cells in a 6-well plate. Colonies were stained with crystal violet and counted by varied sizes (i.e. 100–300 um or > 300 um). Images of wells were captured by Zeiss light microscope, representing one of the three independent biological replicates. (**D**) PC-3 stable cells were seeded in 6-well plates and incubated for 16 hours for wound healing assays. Area of migration measured using Zen imaging software. Cell invasion ability of PC-3 cell lines was measured using matrigel invasion chambers. All results are presented as the mean ± SEM (one-way ANOVA; *n* = 3; ***denotes *p <* 0.001, **denotes *p <* 0.01, and *denotes *p* < 0.05.).

Under 2D culture conditions, BrdU incorporation assays demonstrated that enhanced expression of MEAF6-1 but not MEAF6-2 in both LNCaP and PC-3 cells induced a 40–50% increase of cell proliferation rates (Figure 3A). Since NEPC cells lose their epithelial morphology and favor 3D growth conditions, we also seeded MEAF6-1 and −2 stable lines into matrigel to allow for the formation of multi-cellular spheroids and then performed BrdU incorporation assays. We repeatedly observed that MEAF6-1 stimulated cell proliferation (Figure 3B). MEAF6-1 but not MEAF6-2-overexpressed LNCaP cells also formed significantly larger sizes and numbers of anchorage-independent colonies than control cells (Figure 3C). Furthermore, MEAF6-1 enhanced the migration and invasion rates of PC-3 cells (Figure 3D). Collectively, these results demonstrate that MEAF6-1 and MEAF6-2 have different biological functions in promoting prostate cancer cell proliferation and invasion that can accelerate tumor progression.

### MEAF6-1 transcriptome in prostate cancers

**Figure 4:**
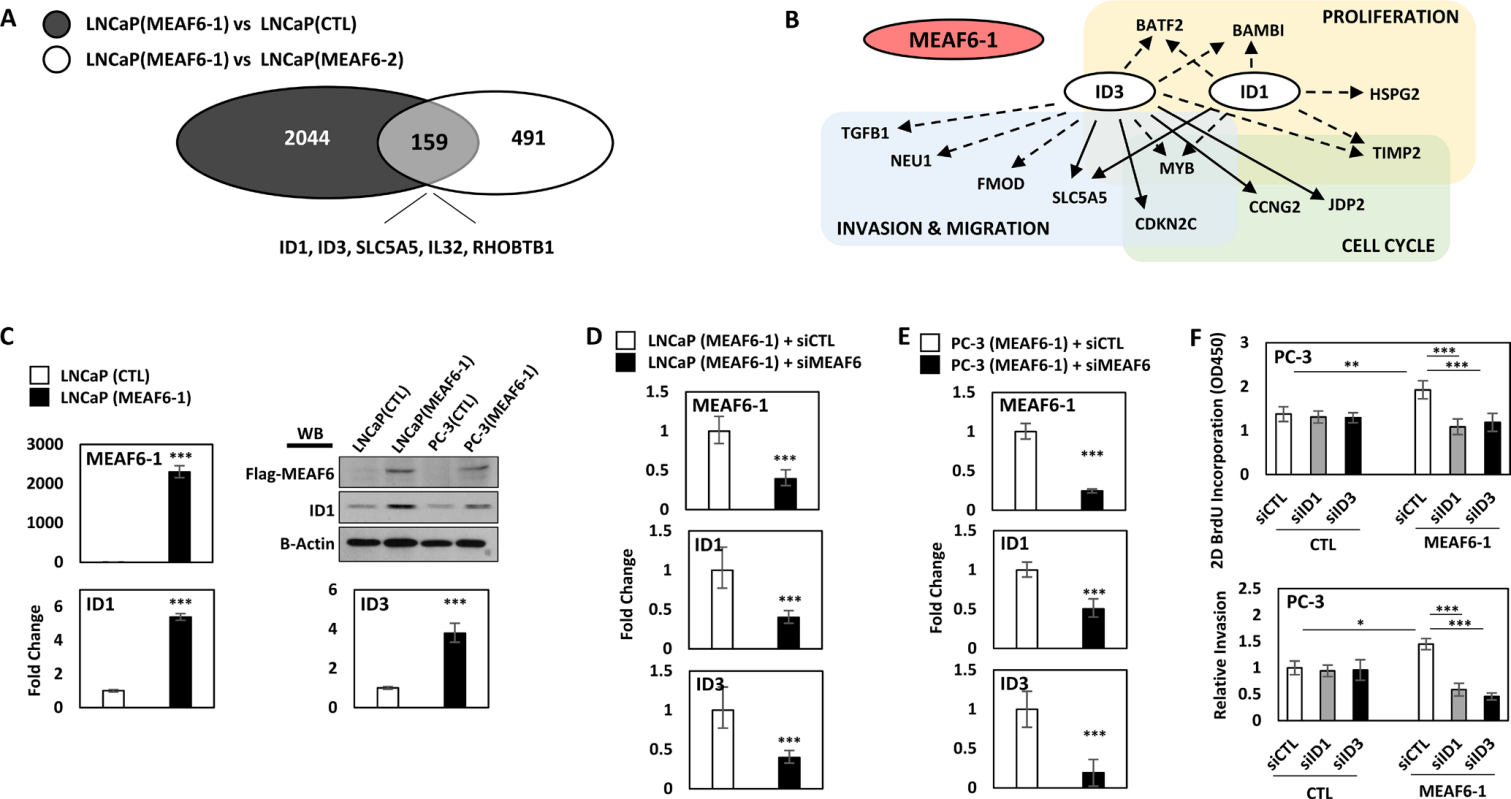
MEAF6-1 function is mediated through ID1 and ID3 Microarray was performed comparing LNCaP(CTL), LNCaP(MEAF6-1), and LNCaP(MEAF6-2). (**A**) Gene expressions with fold changes over 1.3 and an adjusted *p-value* of less than 0.05 from LNCaP(MEAF6-1) vs LNCaP(CTL) were overlapped with LNCaP(MEAF6-1) vs LNCaP(MEAF6-2) transcriptomes. (**B**) LNCaP(MEAF6-1) vs LNCaP(CTL) transcriptomes were uploaded and analyzed in IPA (Ingenuity Pathway Analysis) for Pathway prediction algorithms of ID1 and ID3. Dashed arrows represent indirect regulation, where solid arrows represent direct regulation. (**C**) MEAF6-1-regulation of ID1 and ID3 expression was then validated via real-time qPCR. ID1 protein expression was validated by Western blot. (**D**) LNCaP(CTL) and LNCaP(MEAF6-1) or (**E**) PC-3(CTL) and PC-3(MEAF6-1) stable lines were transfected with siRNA targeting MEAF6 (siMEAF6) to study the effect of MEAF6-1 on ID1 and ID3 expression. Relative quantification of MEAF6-1, ID1, and ID3 compared to 18S via real-time qPCR. (C-E) All results are presented as the mean ± SEM (Student *t-test* ***denotes *p <* 0.001). (**F**) PC-3(CTL) and PC-3(MEAF6-1) stable cells were transfected with control (siCTL), ID1 (siID1), or ID3 (siID3) targeted siRNA. 2D BrdU cell proliferation assays were performed, where BrdU results represent colorimetric quantitative measurements (optical density, OD, at 450 nm wavelength) of BrdU incorporation into DNA. Cell invasion ability of the PC-3 stable cells with siRNA transfections were measured using matrigel invasion chambers. All results are presented as the mean ± SEM (one-way ANOVA; *n* = 3; ***denotes *p <* 0.001 and *denotes *p <* 0.05).

To profile the MEAF6-1 transcriptome, we compared gene microarrays using LNCaP(CTL), LNCaP(MEAF6-1), and LNCaP(MEAF6-2) stable lines. There were 2044 genes differentially regulated by MEAF6-1 and 2702 genes by MEAF6-2. DAVID (Database for Annotation, Visualization, and Integrated Discovery) for gene ontology (GO) analyses indicated that the functions of both gene groups regulated by MEAF6-1 or MEAF6-2 are very similar. GO terms indicated that MEAF6-1 and MEAF6-2-regulated genes are associated with cell cycle, cellular response to stress, cell division, regulation of cell cycle, and DNA replication. Since MEAF6-1, but not MEAF6-2, promotes cell proliferation, anchorage-independent growth and invasion, we further stratified a group of 159 genes that were specifically regulated by MEAF6-1 (Figure 4A). Interestingly, ID1 and ID3 genes are the genes most upregulated by MEAF6-1. When the MEAF6-1-regulated gene list was analyzed by IPA (Ingenuity Pathway Analysis), it predicted that ID1 and ID3 gene networks were the most prominent downstream effectors for MEAF6-1 and are highly associated with cell proliferation, cell cycling, and cell migration and invasion functions (Figure 4B). These microarray results on MEAF6-1 transcriptome were further validated by our real-time qPCR assays showing that MEAF6-1 induced a 5-fold increase of ID1 and a 4-fold of increase of ID3 mRNA levels in LNCaP cells (Figure 4C). Moreover, MEAF6-1 increased protein expression levels of ID1. ID3 protein levels were not validated because of the poor quality of antibodies available against it. Additionally, MEAF6-1-induced ID1 and ID3 expression in LNCaP and PC-3 cells was dramatically compromised when cells were challenged by MEAF6 siRNA (Figure 4D–4E). To further confirm that ID1 and ID3 mediated MEAF6-1 functions in cell proliferation and invasion, we silenced ID1 or ID3 with siRNA and observed that ID1 and ID3 depletion significantly attenuated MEAF6-1 actions on cell proliferation and invasion (Figure 4F). These results indicate that ID1 and ID3 genes play important roles in mediating MEAF6-1 actions for cancer cell proliferation and invasion.

### MEAF6-1 accelerates xenograft tumor growth

**Figure 5:**
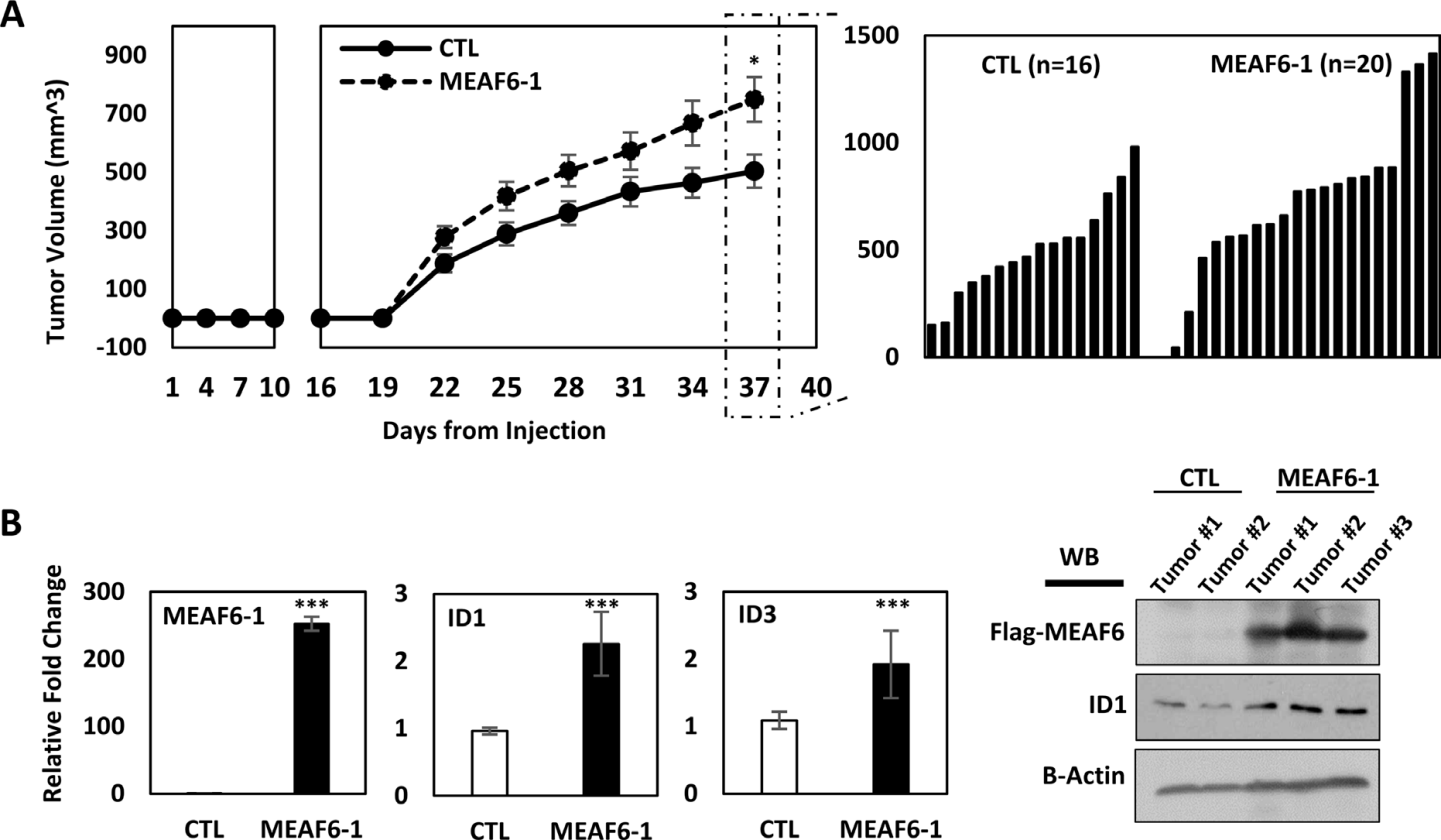
MEAF6-1 accelerates xenograft tumor growth (**A**) 1 × 10^6^ PC-3(CTL) or PC-3(MEAF6-1) were subcutaneously injected into nude mice (CTL *n* = 16; MEAF6-1 *n* = 20). Tumor volume was measured using a caliper. (**B**) Tumors were extracted and processed for RNA and protein analysis. Relative quantification of MEAF6-1, ID1, and ID3 compared to 18S via real-time qPCR. Protein expressions was detected via Western blot using antibodies against exogenous Flag-MEAF6, ID1, and loading control beta-actin. Results are presented as the mean ± SEM (Student *t-test*; ***denotes *p* < 0.001 and *denotes *p* < 0.05).

To study the impacts of MEAF6-1 on xenograft growth, we transplanted PC-3(CTL) or PC-3(MEAF6-1) cells subcutaneously into nude mice. Tumor uptake at ~3 weeks was similar in both groups. However, tumor volume of PC-3(MEAF6-1) xenografts increased significantly faster than control tumors (Figure 5A). Mice were sacrificed before tumor burden reached humane points and xenograft tissues were collected. Real-time qPCR and immunoblotting assays validated exogenous MEAF6-1 expression in PC-3(MEAF6-1) xenografts as well as MEAF6-1 upregulation of ID1 mRNA and protein and ID3 mRNA levels (Figure 5B). Together with the results from Figures 3, 4, these results confirm that MEAF6-1 promotes prostate xenograft growth in part through ID1 and ID3 genes.

## DISCUSSION

**Figure 6:**
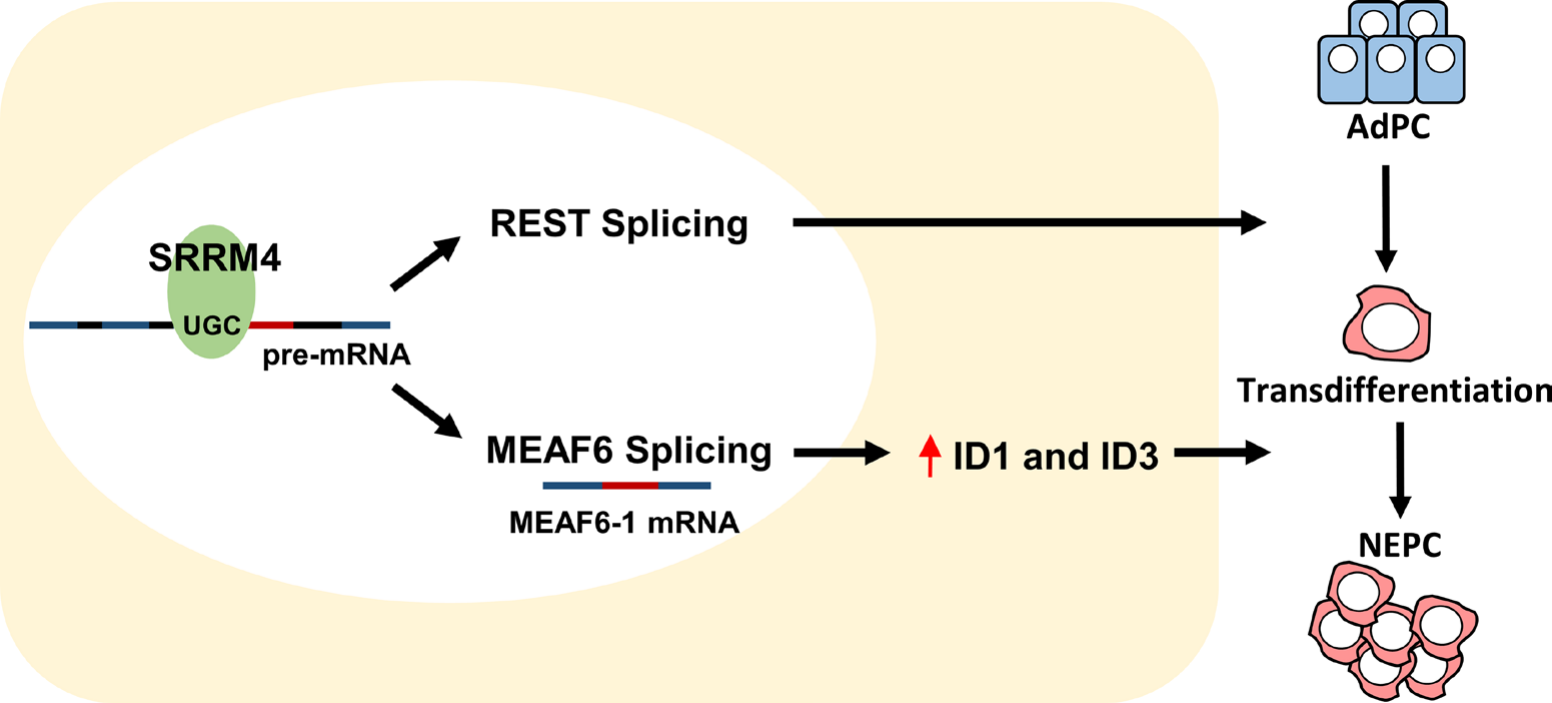
Differentiation and proliferation: two distinguishable and coordinated processes for AdPC to NEPC tumor establishment SRRM4 re-directs MEAF6 functions via alternative RNA splicing to stimulate tumor cell proliferation and invasion through ID1 and ID3.

This study reports that a functionally reprogrammed MEAF6 gene alternatively spliced by neuronal splicing factor SRRM4 facilitates NEPC progression. Spliced by SRRM4, the MEAF6-1 variant does not mediate neuroendocrine differentiation of prostate cancer cells, but rather promotes cell proliferation and invasion to accelerate tumor growth. MEAF6-1 exerts these actions mainly via the ID1 and ID3 genes, which are well characterized inhibitors of cell differentiation as well as enhancers of cell proliferation and multipotency preservation [26]. These findings highlight the critical role of alternative RNA splicing in NEPC progression and support SRRM4 as a master driver of t-NEPC. Through RNA splicing, SRRM4 simultaneously reprograms REST functions to confer a neuroendocrine phenotype in AdPC cells and re-directs MEAF6 functions to stimulate tumor cell proliferation and invasion (Figure 6).

To our knowledge, this is the first report that characterizes the biological functions of the MEAF6 gene with a particular focus on the actions of MEAF6 gene splicing and its role in NEPC progression. It is known that MEAF6 is a component of 4 different MYST family of HAT complexes in cells that modulate gene transcription through posttranslational modifications of histones [20]. However, no studies have confirmed whether MEAF6 can exert enzymatic activities directly to acetylate histones or whether it serves as an adapter to recruit differential protein factors to the HAT complexes. It is not clear whether or not MEAF6 can regulate transcription of endogenous genes. The existence of this gene in the HAT complex has only been confirmed by mass spectrometry; however, this method cannot differentiate between the different splice variants of MEAF6 [27]. Our studies show that the MEAF6-2 variant is ubiquitously expressed in prostate cancer cells, while MEAF6-1 is specifically expressed in NEPC. While MEAF6-2 contains similar transcriptomes as MEAF6-1, it does not affect prostate cancer cell differentiation, proliferation, or invasion. These findings emphasize that the MEAF6-1-specific transcriptome is more important for prostate cancer proliferation, invasion, and tumor growth. The addition of 10 amino acids in MEAF6-1 is regulated by SRRM4 and results in 159 genes that are differentially regulated by MEAF6-1. These results are consistent with the report that SRRM4 commonly targets “microexons” to modulate protein-interaction networks during neurogenesis, where deregulation of microexon-mediated networks are associated with brain disorders such as autism [28]. Since SRRM4 and MEAF6-1 expression is NEPC specific, we propose that functional reprogramming of the MEAF6 gene by SRRM4 contributes to NEPC progression by accelerating proliferation of AdPC cells that have acquired neuroendocrine phenotypes.

We report that MEAF6-1 facilitates NEPC progression mainly by activating the ID1 and ID3 gene networks. The ID family of proteins are inhibitors of bHLH, ETS, and PAX transcription factors and RB family members which regulate cell differentiation [29]. ID protein expression is high in stem and progenitor cells and, while often downregulated during normal cell differentiation, are reactivated in cancer [26]. While tumor suppressor genes such as RB1 and TP53 inhibit ID proteins from activation [30, 31], excessive ID proteins override the tumor suppressor activities of RB1 [32]. Therefore, MEAF6-1-induced ID1 and ID3 may create a condition mimicking genetic inactivation of RB1 and TP53. In this context, MEAF6-1 plays a similar role as SV40 in the TRAMP mouse model, where prostatic specific expression of SV40 sequesters RB1 and TP53 from activation and leads TRAMP tumor progression into NEPC [33, 34]. As a result, our findings define a SRRM4-MEAF6-1-RB1/TP53 axis that contributes to NEPC progression.

In summary, our studies demonstrate that SRRM4-mediated MEAF6-1 splicing is a facilitator for NEPC progression.

## MATERIALS AND METHODS

### Sample collection, RNA-sequencing, and bioinformatics analyses

VPC and Beltran cohort RNA-seq data was previously analyzed and published [18, 19]. RNA samples from patient-derived xenograft were reported previously [4].

### Cell lines and cell culture

LNCaP, C4-2, PC-3, 22Rv1, DU145, NCI-H660, and VCaP cell lines were purchased from American Type Culture Collection (ATCC; Manassas, VA, USA). LNCaP95 (aka LN95) cells were a generous gift from Dr. Alan Meeker of Johns Hopkins University. Small cell lung cancer (SCLC) cells, NCI-H69 and -H82, were generously provided by Dr YZ Wang from the Vancouver Prostate Centre. 293T cells were generously provided by Dr. Paul Rennie and Dr. Ralph Buttyan from the Vancouver Prostate Centre. VCaP, PC-3, DU145 and 293T cells were cultured in DMEM with 10% fetal bovine serum (FBS), whereas LNCaP and 22Rv1 cells were cultured in RPMI-1640 medium with 10% FBS. LNCaP95 was maintained in phenol-free RPMI-1640 medium with 10% charcoal-stripped serum (CSS) (Hyclone). NCI-H660 was cultured in HITES medium (RPMI-1640 medium containing 0.005 mg/ml Insulin, 0.01 mg/ml Transferrin, 30 nM Sodium selenite, 10 nM Hydrocortisone, 10 nM beta-estradiol, and 2 mM L-glutamine) with 10% FBS. NCI-H69 and NCI-H82 suspension cells were maintained with RPMI-1640 medium supplemented with 10% FBS, 100 ug/ml streptomycin, and 100units/ml penicillin. Cells were all incubated in 5% CO_2_ at 37C.

### Real-time qPCR and Western blotting

Real-time qPCR assays were performed as previously described [4, 35–37]. Assays were carried out using three technical replicates and three independent biological replicates. Primer information is listed in Supplementary Table 1. Western blotting assays were performed as reported [4, 35–37]. All Western blot experiments were repeated in three independent experiments with one representative blot shown. Antibody information is listed in Supplementary Table 2.

### DNA and siRNA transfections

Cells were transfected with Silencer Select (Ambion) siRNA targeting ID1 (siRNA ID: s7106) or ID3 (siRNA ID: s7110), or with Silencer Select Negative Control No. 1 siRNA (Ambion) using Lipofectamine 3000 (Invitrogen) according to manufacturer's protocol. Lipofectamine 3000 was also used for transient DNA plasmid transfections. Flag-SRRM4 expression vector was previously described [4].

### Construction of expression plasmids and prostate cancer cell lines by lentiviral approaches

MEAF6-1 and MEAF6-2 cDNA (Integrated DNA Technologies) were cloned into pCMV2 expression vector as previously described [35]. Lentiviral expression vectors encoding Flag-MEAF6-1 or Flag-MEAF6-2 were created as described previously [4, 35]. Vectors were used to package lentivirus and infect LNCaP and PC-3 cells. All cell lines were cultured under blasticidin selection (LNCaP, 5 ug/ml; PC-3, 10 ug/ml). MEAF6-1 and MEAF6-2 expression was confirmed by real-time qPCR and immunoblotting assays (Supplementary Figure 2). The fidelity of all PCR-generated fragments and expression plasmids were confirmed by DNA sequencing and expressed proteins were validated using anti-flag antibody before and after application in construction of stable lines.

### RNA-ChIP assays

RNA-ChIP assays followed the protocol as previously published [4, 35]. Briefly, LNCaP and 293T cells were transfected with mock (control) or Flag-SRRM4 plasmid. RNA-protein complex was cross-linked with formaldehyde and immunoprecipitated with anti-Flag antibody. Eluted RNA fragments were used as templates for real-time qPCR. Data was calculated as a percentage of input. Primer sequences are listed in Supplementary Table 1.

### Cell proliferation, migration, invasion, and colony formation assays

Cell proliferation, migration, and invasion assays were described previously [4, 36]. Briefly, 2D and 3D cell proliferation was measured with the bromodeoxyuridine (BrdU) proliferation assay kit (Millipore, Catalogue # 2750) according to manufacturer's protocol and as previously described [4]. Cells were seeded in matrigel for 3D BrdU proliferation assays. For migration assays, a monolayer wound was created when cells reached 100% confluency. Cell migration was subsequently captured at time point 0h and 24h after wound scratch. Migration ability of cells were calculated as the migration distance from 0h to 24h. Cell invasion assays were carried out by using BD BioCoat Matrigel Invasion chambers (BD Biosciences) according to manufacturer's protocol. Invasion rate were calculated as the percentage of cell invasion through the Matrigel. In the colony formation assays, about 2 × 10^4^ cells were seeded in 0.7% soft agar in a 6-well plate, with a 1% soft agar bottom base coating. Cells were allowed to grow for 14 days to form colonies. Colonies were stained with crystal violet and imaged by stitching 5X field images together to capture the entire well (Zeiss light microscope [Carl Zeiss]). Colony numbers were counted according to their diameters (100–300 um or > 300 um). Three independent biological replicates were performed for all the assays.

### Gene microarray

Gene microarray methods were described previously in detail [36]. In summary, two independent repeated experiments of LNCaP(CTL) and three independent repeated experiments of LNCaP(MEAF6-1) and LNCaP(MEAF6-2) cells (a total of 8 samples) were extracted for total RNA using the mirVana RNA Isolation Kit (Ambion) according to manufacturer's protocol and subsequently hybridized to human GE 8 × 60 K gene expression microarray (Agilent). The resulting data was processed by the Agilent Feature Extraction software. For statistical analyses, comparisons were performed between RNA samples with LNCaP(CTL) *vs* LNCaP(MEAF6-1), LNCaP(CTL) *vs* LNCaP(MEAF6-2), and LNCaP(MEAF6-1) *vs* LNCaP(MEAF6-2). Unpaired *t-test* with *P value* cut-off of 0.05, a fold change cut-off of 1.5, and Benjamini-Hochberg multiple testing correction were applied. LNCaP(CTL) *vs* LNCaP(MEAF6-1) transcriptomes were then uploaded and analyzed in IPA (Ingenuity Pathway Analysis) for Pathway prediction algorithms.

### Human prostate cancer xenografts

To construct PC-3 xenografts, each PC-3 cell line (1 ×10^6^ cells per line) was implanted subcutaneously in bilateral flanks of 6–8-week old male nude mice (Nu/Nu). Tumor volume (V = length * width * high * 0.5236) was measured three times a week. All animal procedures were under the guidelines of the Canadian Council on Animal Care.

### Statistics

All results are expressed as the mean ± SEM. One-way ANOVA or Student *t-test* was carried out using GraphPad Prism (version 6) to determine differences between groups with the level of significance set at *p <* 0.05 as *p < 0.01 as **, and *p <* 0.001 as ***.

## SUPPLEMENTARY MATERIALS FIGURES AND TABLES


