# Supplementary material for: Alternative RNA splicing of the MEAF6 gene facilitates neuroendocrine prostate cancer progression
Source: Oncotarget. 2017 Mar 2;8(17):27966–75. doi: 10.18632/oncotarget.15854 (PMC5438622; doi:10.18632/oncotarget.15854)
Supplement: Supplementary file 1 [file oncotarget-08-27966-s001.pdf]

Alternative RNA splicing of the MEAF6 gene facilitates neuroendocrine prostate cancer progression

Supplementary Materials

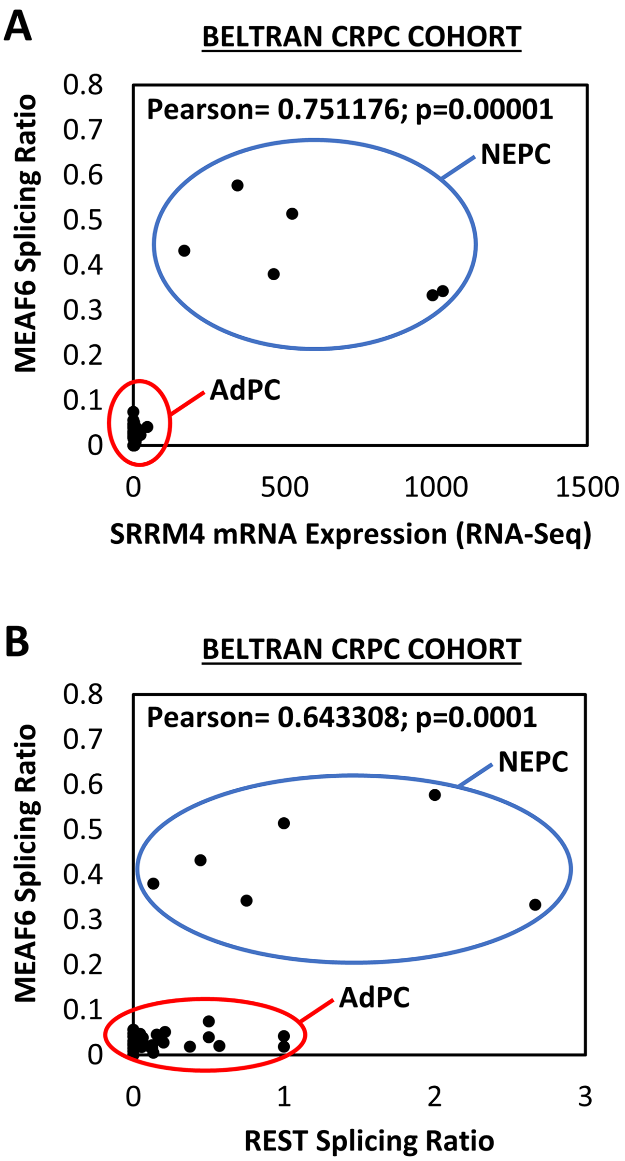

Supplementary Figure 1: MEAF6 RNA splicing is positively correlated with SRRM4 expression and REST RNA splicing. Pearson's *r* correlation coefficient between MEAF6 splicing ratio (MEAF6-1:MEAF6-2 RNA-seq reads per base-pair) and (**A**) SRRM4 total expression or (**B**) REST splicing ratio (REST4:REST RNA-seq reads per base-pair) obtained from the Beltran CRPC patient RNA-seq dataset (NEPC $n = 6$ and AdPC $n = 32$).

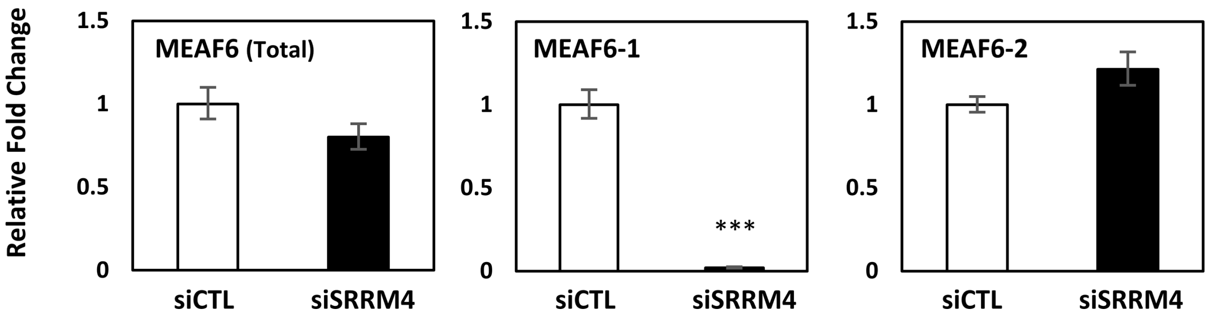

Supplementary Figure 2: SRRM4 regulates RNA splicing of MEAF6. LNCaP(SRRM4) stable cells were seeded in 6-well petri dishes in RPMI1640 medium with 10% fetal bovine serum (FBS) and transfected the next day. Cells were transfected with 20 uM of control or SRRM4-targeted siRNA for 48 hours and then collected for RNA extraction for real-time qPCR validation. Relative quantifications of total MEAF6, MEAF6-1, and MEAF6-2 were compared to 18S.

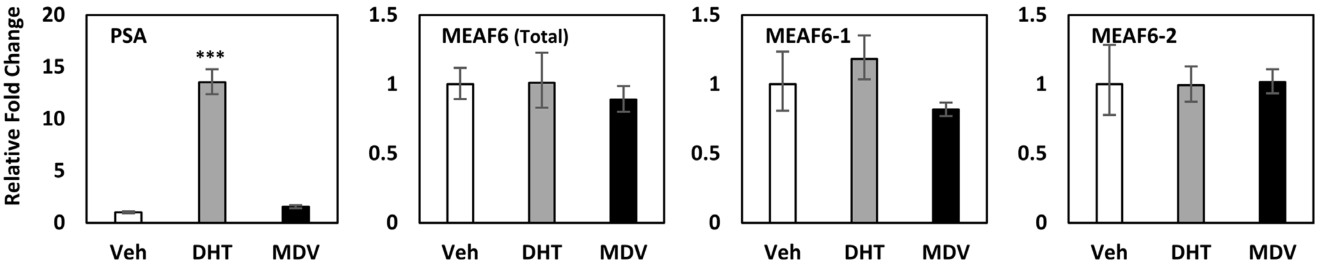

Supplementary Figure 3: Androgen receptor signaling does not regulate MEAF6 variant expression. LNCaP cells were cultured in phenol-free RPMI-1640 medium with 5% charcoal-stripped serum (CSS) for 48 hours. Cells were then treated with DMSO (vehicle), 10 nM AR agonist DHT (dihydrotestosterone; Cedarlane), or 5 uM AR antagonist MDV3100 (Enzalutamide; Haoyuan Chemexpress) for 24 hours and subsequently collected for RNA extraction for real-time qPCR. Relative quantification of total MEAF6, MEAF6-1, MEAF6-2, and PSA were compared to 18S. All results are presented as the mean ± SEM (Student *t*-test; $n = 3$, ***denotes $p < 0.001$). AR, androgen receptor; PSA, prostate specific antigen.

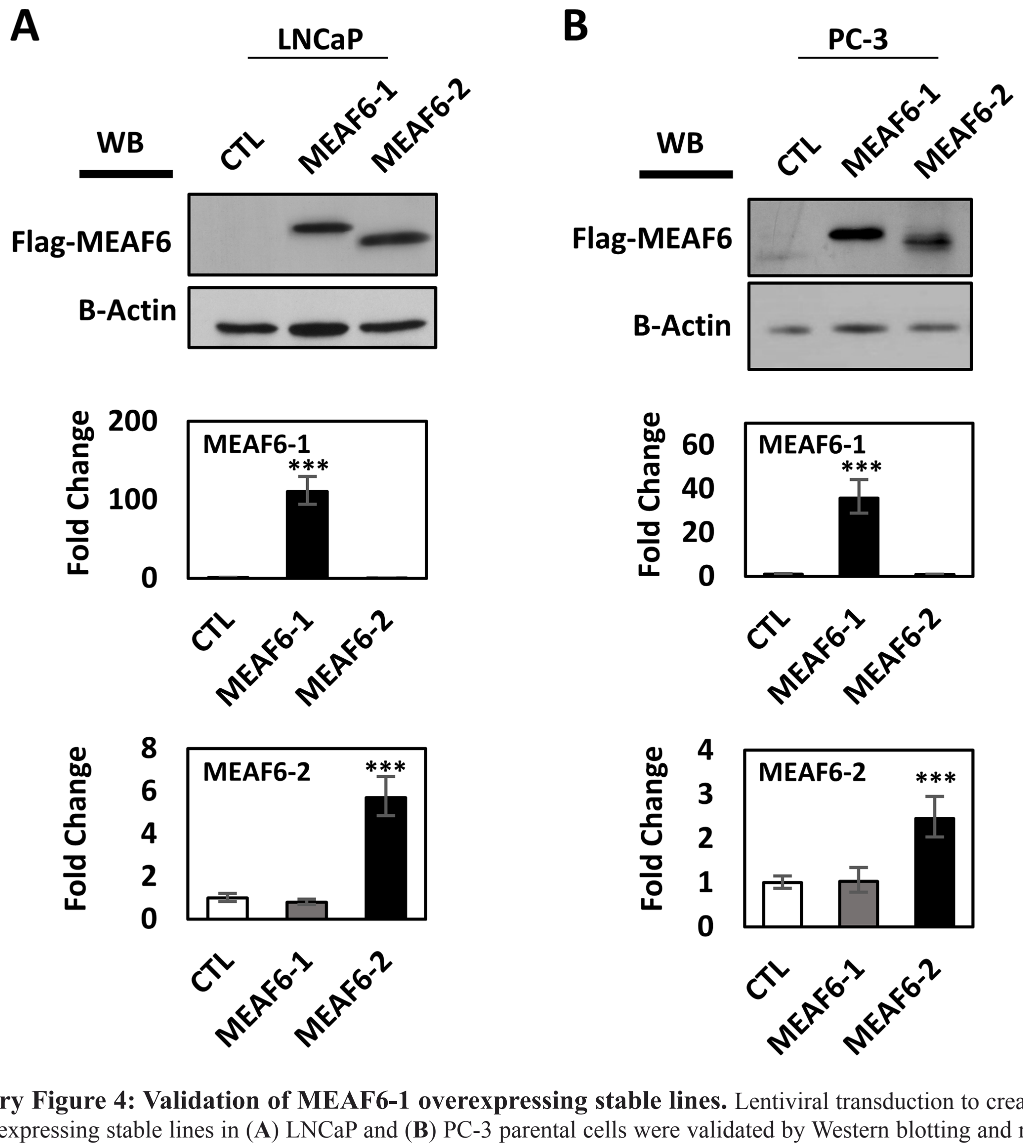

Supplementary Figure 4: Validation of MEAF6-1 overexpressing stable lines. Lentiviral transduction to create MEAF6-1 or MEAF6-2 overexpressing stable lines in (**A**) LNCaP and (**B**) PC-3 parental cells were validated by Western blotting and real-time qPCR. Anti-flag antibody was used to detect exogenous MEAF6-1 and MEAF6-2 expression. Anti-beta-actin antibody was used as a loading control.

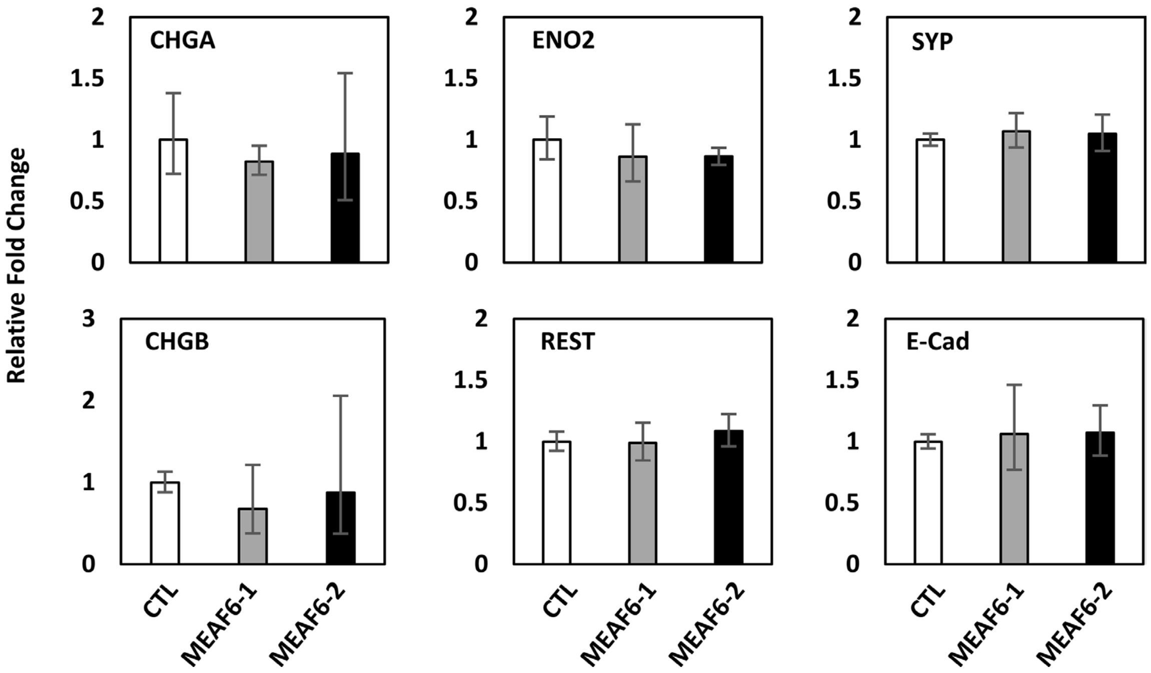

Supplementary Figure 5: MEAF6 does not facilitate neuroendocrine trans-differentiation. LNCaP(CTL), LNCaP(MEAF6-1), and LNCaP(MEAF6-2) cells were collected for total RNA extraction. Expression of neuroendocrine markers (i.e. CHGA, CHGB, ENO2, and SYP) and epithelial markers (i.e. REST and E-Cad) were determined by real-time qPCR. Relative quantifications were compared to 18S.

Supplementary Table 1: Primers for real-time qPCR

Gene Name	Sequence
18S F	5′- TTG ACG GAA GGG CAC CAC CAG -3′
18S R	5′- GCA CCA CCA CCC ACG GA A TCG -3′
MEAF6-1 F	5′- GAA TAA AAA CCG GCA CAG CCC -3′
MEAF6-2 F	5′- GAA TAA AAA CCG GCA CAG GAT TG-3′
MEAF6-1 & MEAF6-2 R	5′- CTA ATA GTC AGC TCG TGG TTT TTT G -3′
MEAF6 Total F	5′- AGG AGC TGG CGG AAA CAT TG -3′
MEAF6 Total R	5′- TGG TTG GTC AGA TAC CGA TCC -3′
SRRM4 F	5′- CAC AAG CGA CGC AGG TCA T -3′
SRRM4 R	5′- CGG TGG CGG TGA GAC TTT C -3′
MEAF6 RNA-CHIP F	5′- ACC AAT TCT TGC TAG GGT GGC TA -3′
MEAF6 RNA-CHIP R	5′- TCA TAA TCA AAC ATG CCA GAC G -3′
GAPDH RNA-CHIP F	5′- GGC ATG GAC TGT GGT CAT GAG -3′
GAPDH RNA-CHIP R	5′- TGC ACC ACC AAC TGC TTA GC -3′
ID1 F	5′- TCT GCA CAC CTA CTA GTC ACC A -3′
ID1 R	5′- GAG AAG CAC CAA ACG TGA CC -3′
ID3 F	5′- GGA GCG AAG GAC TGT GAA CT -3′
ID3 R	5′- CCA CGC TCT GAA GAG ACC TT -3′
CHGA F	5′- TAA AGG GGA TAC CGA GGT GAT G -3′
CHGA R	5′- TCG GAG TGT CTC AAA ACA TTC C -3′
CHGB F	5′- CGA GGG GAA GAT AGC AGT GAA -3′
CHGB R	5′- CAG CAT GTG TTT CCG ATC TGG -3′
ENO2 F	5′- CCG GGA ACT CAG ACC TCA TC -3′
ENO2 R	5′- CTC TGC ACC TAG TCG CAT GG -3′
E-Cad F	5′- ATT TTT CCC TCG ACA CCC GAT -3′
E-Cad R	5′- TCC CAG GCG TAG ACC AAG A -3′
SYP F	5′- TTA GTT GGG GAC TAC TCC TCG -3′
SYP R	5′- GGC CCT TTG TTA TTC TCT CGG TA -3′
PSA F	5′- AGT GCG AGA AGC ATT CCC AAC -3′
PSA R	5′- CCA GCA AGA TCA CGC TTT TGT T -3′

Supplementary Table 2: Antibodies

Antibody	Clone ID	Cat No.	Supplier
Flag	M5	F4042	Sigma Aldrich
Beta-actin	C-11	sc-1615	Santa Cruz Biotech
ID1		Ab134163	Abcam
